# Sleep Strategies and Performance Outcomes in Ultramarathon Runners: A Systematic Review

**DOI:** 10.1177/19417381261494498

**Published:** 2026-10-28

**Authors:** Daniel A. Brown, Jason Parreira, Katie Spencer, Serena Skinner, Wataru Goto, Larissa Sattler

**Affiliations:** † Physiotherapy Department, Gold Coast Hospital and Health Service, Gold Coast, Queensland, Australia; ‡ Physiotherapy Department, Bond University, Gold Coast, Queensland, Australia; § Doctor of Physiotherapy Student, Bond University, Gold Coast, Queensland, Australia; ‖ Griffith University, School of Allied Health, Sport and Social Work, Parklands Drive, Southport Gold Coast Queensland, Australia

**Keywords:** cognitive performance, endurance, intra-race sleep, sleep deprivation, sleep extension

## Abstract

**Context:**

Ultramarathon events impose prolonged physiological and cognitive demands, often spanning 1 or more nights with limited sleep opportunity.

**Objective:**

To examine associations between pre-race and intra-race sleep strategies and physical and cognitive performance outcomes in ultramarathon runners.

**Data Sources:**

PubMed, EMBASE, PsycINFO, SPORTDiscus, and Web of Science were searched from inception to July 25, 2025.

**Study Selection:**

Primary observational or experimental studies published in English, or available in English translation were eligible when they involved ultramarathon runners (>42.2 km) and reported sleep as a variable of interest in relation to race performance or cognitive performance.

**Study Design:**

Systematic review.

**Level of Evidence:**

Level 3.

**Data Extraction:**

Two reviewers independently extracted study design, participant characteristics, race characteristics, sleep outcomes, performance outcomes, and cognitive outcomes. Methodological quality was assessed using the Joanna Briggs Institute critical appraisal checklists.

**Results:**

Nine studies involving 2262 participants were included, with overall low risk of bias. Of the 4 studies examining pre-race sleep, 3 reported a significant association or group difference with a race-performance outcome. Reduced sleep was generally associated with poorer physical and cognitive performance. Greater intra-race sleep duration was consistently associated with longer finish times, reflecting additional nonmoving time and increased sleep need in longer events. Greater pre-race sleep duration, including sleep extension, was associated with faster finish times, and better pre-race sleep quality was linked to higher cognitive performance.

**Conclusion:**

Pre-race sleep extension was associated with faster finish times, although the observational evidence does not establish causality. Further research using objective sleep measures and controlled designs is required.

Participation in ultramarathon running has increased rapidly in recent years, reportedly rising by 345% between 2012 and 2022.^19,51^ Ultramarathons are defined as running events that exceed the standard marathon distance of 42.2 km.^48^ These events occur in multiple formats, where distance-based races typically include 50 km (31 miles), 80 km (50 miles), 100 km (62 miles), or 161 km (100 miles), while timed events aim to accumulate the greatest distance within a set duration, often 6, 12, or 24 hours.^19,51^ Another format gaining popularity is the Backyard Ultra, in which runners complete a short loop every hour on the hour until only 1 participant remains, with elimination occurring through voluntary withdrawal or failure to meet the hourly cut-off.^3^ Ultramarathons may also be completed as multi-day continuous or multi-day staged races.^19,51^ Ultramarathons may be run on roads, but they take place more commonly on trails and, at times, through rugged or unmarked terrain.^19^ By requiring prolonged physical exertion across extended durations, often spanning ≥1 night, ultramarathon events test both physiological and psychological limits. The variety of event formats and the appeal of extreme challenge may help explain the recent surge in participation.

Despite the growing research interest in ultramarathon events, the influence of sleep on performance remains poorly understood.^2,37,42^ This gap likely reflects the logistical challenges of studying races that are lengthy, physically demanding, and frequently held in remote environments.^50^ It may also reflect that ultramarathon research has expanded only relatively recently compared with other endurance sports. Furthermore, although poor sleep is known to negatively affect performance in endurance athletes,^38,41,45,47,49^ much less is known about the sleep strategies used by ultramarathon runners and how these strategies relate to performance.^33^ In this review, sleep strategies refer to intentional approaches used to optimize sleep opportunity before a race or to manage sleepiness during a race. These may include pre-race sleep extension, training under sleep restriction, planned naps, and brief intra-race “micro-naps” or short sleep episodes.^33^

In endurance more broadly, the relationship between sleep and performance has been well documented, particularly the impact of sleep quality and sleep duration before competition.^27^ Many athletes report disturbed sleep in the lead-up to events. For example, Lastella et al^28^ found that endurance cyclists experienced their poorest sleep quality on the night before competition, with almost 60 minutes less total sleep compared with baseline. Proposed contributors include variable start times, travel across time zones, and heightened precompetition arousal such as anxiety or excitement.^28^ Similar findings have been reported in other athletic populations, including elite Australian athletes, among whom 64% reported sleep issues the night before competition.^24^

Sleep management during ultra-endurance events appears to be highly variable, and may depend on event structure and duration.^30^ Léger et al^30^ examined sleep during a multistage sailing race and found that sleep management was difficult, with participants accumulating substantial sleep debt and spending more time sleeping during competition rather than between stages. The authors also reported that teams with better race outcomes demonstrated more effective sleep management during the event and invested more in pre-race sleep preparation. Although sailing differs from ultramarathon running, both contexts can involve prolonged exertion across ≥1 night, time pressure, and sustained cognitive demands, which makes this evidence useful for framing why sleep strategies may matter in ultramarathon events.^30^

Athletes also use a range of strategies to increase sleep opportunity during training and competition periods. Wilson and Baker^52^ reported that elite athletes often used naps and earlier bedtimes to increase time available for sleep, but also tended to show lower sleep efficiency, meaning more time in bed was spent attempting to sleep rather than sleeping.^52^ Napping has been associated with improvements in endurance performance, including in runners.^4^ At the same time, high training volumes can adversely affect sleep quality and contribute to performance impairment.^17^ Athletes also commonly report poor sleep quality as a feature of persistent fatigue, with fatigue associated with reduced performance.^35^ In addition, sleep deprivation combined with prolonged physical exertion can impair cognitive function.^11^ This is relevant to ultramarathons because impaired cognition and vigilance may have safety implications, particularly in remote settings or on technical terrain that requires sustained attention and decision-making. Notably, sleep deprivation appears to impair performance regardless of whether athletes are untrained, recreationally trained or well-trained.^31^

To our knowledge, no systematic review has specifically synthesized evidence on sleep strategies used by ultramarathon runners, such as pre-race sleep extension and intra-race sleep (including naps and micro-naps), and their associations with performance outcomes. Understanding these relationships may enable runners and coaches to develop more targeted preparation, recovery, and race-day planning. It may also support participant safety by improving anticipation, recognition, and management of sleep deprivation signs and symptoms before or during events. Therefore, this systematic review aimed to examine the associations between pre-race and intra-race sleep strategies and physical and cognitive performance outcomes in ultramarathon runners.

## Methods

This systematic review was developed and reported in accordance with the Preferred Reporting Items for Systematic Reviews and Meta-Analyses (PRISMA).^39^ The protocol was registered with the Open Science Framework (OSF) (http://osf.io/b7esd).

### Eligibility Criteria

The review question was developed using the Population, Exposure, Outcomes (PEO) framework to identify all available evidence on sleep in ultramarathoners^20^:

- Population: runners participating in an ultramarathon event (distance >42.2 km);
- Exposure: sleep strategies or behaviors before or during an ultramarathon event;
- Outcomes of interest: performance during the ultramarathon event (time or distance) or cognitive performance.

In addition to the PEO criteria listed above, the inclusion criteria were as follows:

- Published as full-text articles in English, or available in English translation;
- Primary observational or experimental research;
- Conducted in human participants of any age, sex, or geographic region.

The exclusion criteria were as follows:

- Running events ≤42.2 km;
- Nonrunning populations or nonrunning events;
- No sleep-related exposure or variable of interest;
- Unavailable full text.

### Information Sources and Search Strategy

A comprehensive search was conducted with no date restrictions from database inception to July 25, 2025 in the following databases: PubMed, EMBASE, PsycINFO, SPORTDiscus, and Web of Science. A preliminary search was undertaken in PubMed using key terms including “ultramarathon,” “ultramarathon runners,” “sleep strategies,” and “performance.” This preliminary search was used to identify additional relevant terms and synonyms, supported by the TERA Tools Word Freq feature.^8^ The final search strategy used keywords and synonyms combined with Medical Subject Headings and Boolean operators. The search strategy was constructed and peer-reviewed by the Bond University Health Science and Medicine faculty librarian and trialed using PubMed’s advanced search feature. Using the TERA Tools Polyglot Feature, it was translated for use with the other databases.^7,8^ The complete search strategy for each database can be found in Supplementary File 1 (available in the online version of this article).

### Study Selection

Search results were exported to EndNote (Version 21, Clarivate Analytics) for record management,^6^ and duplicates were removed using the TERA Deduplication Tool.^8^ Two reviewers collaboratively undertook title and abstract screening in Screenatron (TERA) of 20 articles to ensure consistency.^15^ All remaining records were screened independently by those reviewers. The same reviewers then assessed the full texts of potentially eligible articles independently, with reasons documented for any exclusions at this stage. Any disagreements were resolved through review by a third author to reach consensus.

### Data Extraction

Data were extracted independently by 2 authors using a standardized extraction table in Microsoft Excel adapted from the Cochrane Collaboration Data Collection Template.^18^ Extracted data included author, year, country, study design, participant characteristics, and sample size. Key outcomes of interest were also extracted, including event characteristics (distance, terrain, duration, elevation, ambient temperature), sleep duration, sleep quality, sleep frequency, cognitive performance, and event performance outcomes. Cognitive performance outcomes were recorded as reported by included studies, such as reaction time, attention, processing speed, and executive function. For continuous outcomes (e.g., sleep duration and event performance outcomes), means and standard deviations were extracted where available.

### Critical Appraisal of Individual Sources of Evidence

Methodological quality was assessed independently by 2 authors using the relevant Joanna Briggs Institute (JBI) Critical Appraisal Checklist for each study design, specifically cohort studies and analytical cross-sectional studies.^36^ Each item was rated as ‘Yes,’ ‘No,’ ‘Unclear,’ or ‘Not applicable.’ Disagreements were resolved by a third author to achieve consensus. Inter-rater reliability was assessed using Cohen’s kappa. Values between 0.81 and 1.00 were interpreted as almost perfect agreement, 0.61 to 0.80 as substantial, 0.41 to 0.60 as moderate, 0.21 to 0.40 as fair, and 0.00 to 0.20 as slight.^26^

### Synthesis of Results

Findings were synthesized using narrative and tabular methods guided by common themes and outcomes identified during data extraction. Studies reporting similar sleep characteristics and outcome measures were grouped to support structured comparison. Due to substantial clinical (differences in populations, exposures, outcomes) and methodological (study design, measurement tools across studies) heterogeneity across studies, meta-analysis was not feasible. Therefore, a narrative synthesis was conducted, with results reported by shared sleep exposures and outcome domains.

## Results

### Study Selection

The initial search of selected databases yielded 675 records, of which 287 were identified as duplicates and removed. The remaining 388 records were screened via title and abstract, leaving 33 eligible for full text review. Of the remaining 33 records, 4 were excluded due to being abstract-only reports, resulting in 29 reports to review against the eligibility criteria. In total, 20 were excluded at this stage: 12 due to sleep not being a variable related to a performance outcome, 3 where data were not collected during an ultramarathon event, 2 were review studies, 1 not meeting the required distance of greater than 42.2 km, 1 being an ongoing clinical trial, and 1 due to it being a thesis document. A final total of 9 studies were included in this review. The results of the study screening and selection process are summarized in Figure 1.

**Figure 1.**
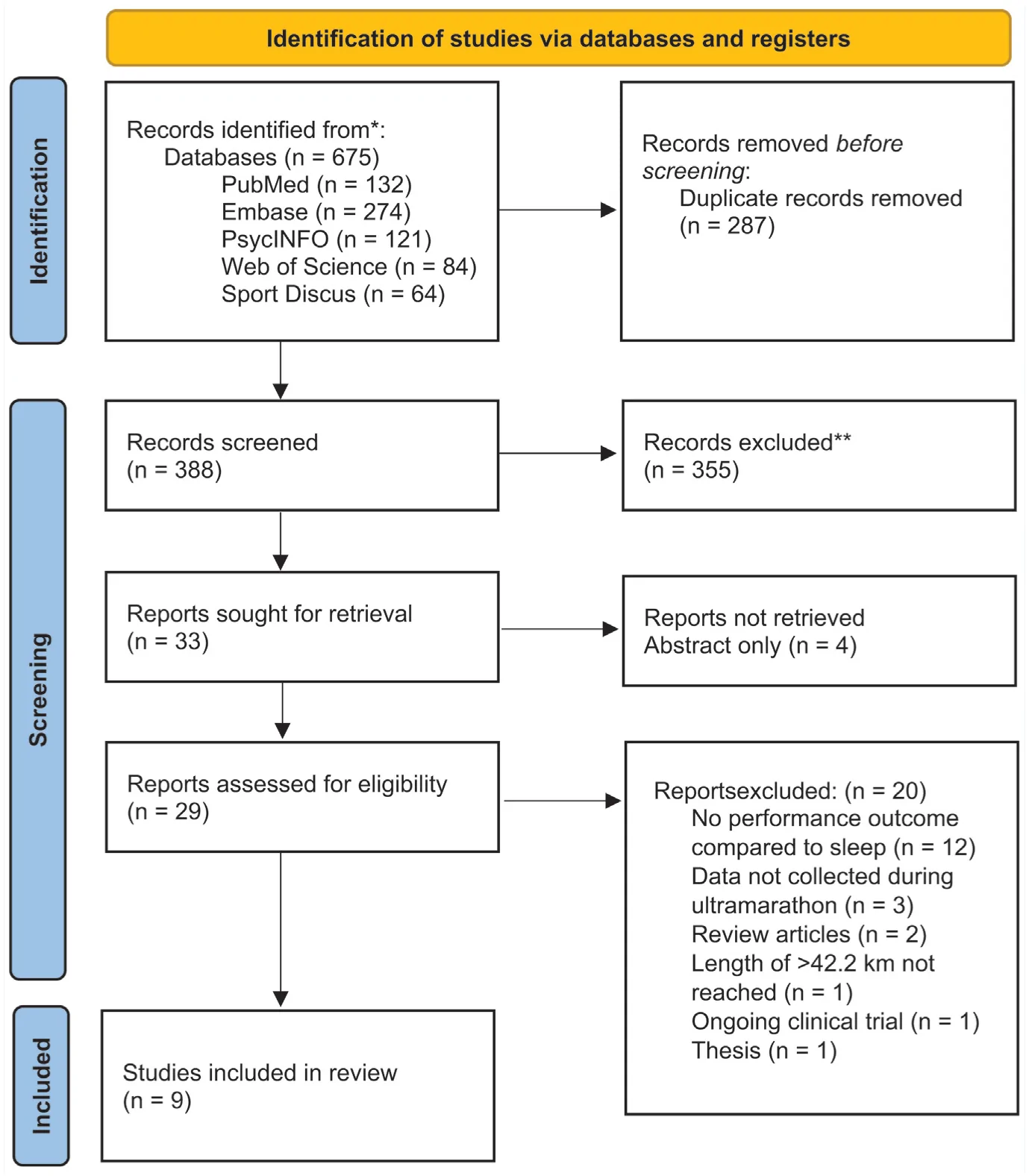
PRISMA flow diagram illustrating the identification, screening, eligibility assessment, and inclusion of studies in the review. PRISMA, preferred reporting items for systematic reviews and meta-analyses.

### Risk of Bias

Overall, the included studies had a low risk of bias with a mean JBI critical appraisal score of 82.4% and an almost perfect level of agreement (k = 0.93) between the 2 reviewers. After discussion with a third reviewer, a second analysis revealed a 100% agreement between reviewers. All the cross-sectional studies failed to mention strategies to deal with confounding variables. Two of the cross-sectional studies did not use a valid and reliable way of measuring sleep or measure fatigue in an objective manner. Supplementary File 2, available online, displays the risk of bias assessment score from the JBI checklist by study type.

### Study Characteristics

Of the 9 included studies, 4 were prospective cohort,^3,12,22,23^ and 5 were cross sectional study designs.^10,13,24,33,40^ These studies yielded 2262 participants comprised of 1683 men (74.4%), 267 women (11.8%), and 312 (13.8%) where sex was not specified. The mean age of the participants included across all studies was 43.7 ± 8.4 years. Individual demographics of the included studies are detailed in Table 1.

**Table 1.** Study characteristics

		Participants				Methodological quality
Author, year, country, study design	Title	Number	Age, years	Male sex, n (%)	Race experience	JBI critical appraisal score (%)
Benchertrit et al,^3^ 2024, UK, prospective cohort	The effects of sleep deprivation and extreme exertion on cognitive performance at the world-record breaking Suffolk Back Yard Ultra-marathon	11	40 ± 8.6	11 (100)	8.6 ± 10.6 races	8 out of 8 (100%)
Daniel et al,^10^ 2024, France and Brazil, cross sectional	Ultramarathon runners and support crew: the influence of pre-race sleep and training profiles on performance in a 217-km mountain race	38	45 ± 8.7	32 (84.2)	13.8 ± 9.1 years	6 out of 8 (75%)
Doppelmayr et al,^12^ 2005, Austria; prospective cohort	Changes in cognitive performance during a 216 km, extreme endurance footrace: a descriptive and prospective study	2	50.5 ± 9.5	2 (100)	17.7 ± 12.5 races	7 out of 9 (78%)
Esteves dos Santos et al,^13^ 2022, Brazil, cross sectional	Sleep and nutritional profile of endurance and ultra-endurance running athletes	9	45.30 ± 8.12	NR	10.18 ± 11.40 years	7 out of 8 (88%)
Hurdiel et al,^22^ 2015, France, prospective cohort	Combined effects of sleep deprivation and strenuous exercise on cognitive performances during The North Face Ultra Trail du Mont Blanc	17	43 ± 6.4	16 (94)	NR*^a^*	11 out of 11 (100%)
Hurdiel et al,^35^ 2018, France, prospective cohort	Cognitive performance and self-reported sleepiness are modulated by time-of-day during a mountain ultramarathon	92	43 ± 7.52	92 (100)	NR*^a^*	11 out of 11 (100%)
Kishi et al,^25^ 2024, France, cross sectional	Sleep and ultramarathon: exploring patterns, strategies, and repercussions of 1154 mountain ultramarathons finishers					
	Race 1, DDF	882	43.1 ± 8.9	774 (87.8)	6.3 ± 6.6 races	6 out of 8 (75%)
	Race 2, TDB	272	41.9 ± 9.1	215 (79.0)	5.6 ± 5.8 races	6 out of 8 (75%)
Martin et al,^33^ 2018, France, cross sectional	Sleep habits and strategies of ultramarathon runners	636	41.6 ± 10.04	541 (85.1)	2.09 ± 1.25 races per year	5 out of 8 (63%)
Poussel et al,^40^ 2015, France, cross sectional	Sleep management strategy and performance in an extreme mountain ultra-marathon	303	44 ± 7.5	NR	Varied (5.68 ± 3.21 years)*^b^*	5 out of 8 (63%)

DDF, Diagonale des Fous; JBI, Joanna Briggs Institute; NR, not reported; TDB, Tour de Bourbon; UK, United Kingdom.

a At least 2 mountain ultramarathons ran in the past 2 years.

b Thirty (10%) <2 years of experience, 121 (44%) between 2 and 5 years experience, 109 (36%) between 5 and 10 years experience, and 30 (10%) >10 years.

The participants of the included studies completed events in various settings, formats and distances; 7 of the included studies involved events in a mountainous environment.^10,12,13,22,23,25,40^ Table 2 outlines the race characteristics of the included studies. Of these events, 3 examined the same race though in different years; the Ultra Trail du Mont-Blanc (UTMB) race.^22,23,40^ One study took place in rolling forest terrain,^3^ and 1 study did not specify terrain type.^33^ Of the included studies, 4 were “point to point” style races where the finish line is at a location separate from the start line,^10,12,13,35^ 3 were loop style courses where the start and finish line are in the same location,^22,23,40^ and 1 was a backyard ultra-format.^3^ The distance covered was variable, ranging from 50 km to 217 km,^10,13,25,40^ with 1 study not reporting total distance.^33^ In addition, total elevation gained across the length of the course was highly varied across all events. Elevation gain was highest in the mountain ultramarathons, with the most in a single race being 10,000 meters of positive elevation gain across the 168 km UTMB course in 2015,^23^ and the lowest being 23 meters of positive elevation gain per lap of the backyard ultramarathon.^3^

**Table 2.** Race characteristics

Author (year)	Type	Distance, km	Terrain	Elevation, meters	Temperature, °C
Benchertrit et al^3^ (2024)	Nonstop backyard ultra-running race	158.5 ± 53.8	Rolling forest	23	10-22
Daniel et al^10^ (2024)	Point to point	217	Mountain	Total positive and negative change of 12,000	NR
Doppelmayr et al^12^ (2005)	Point to point	216	Mountain	80 below, 2500 above	37-53
Esteves dos Santos et al^13^ (2022)	Point to point	50-100	Mountain	NR	NR
Hurdiel et al^22^ (2015)	Loop mountain UM	168 (104 mile)	Mountain	9600 positive altitude change	NR
Hurdiel et al^23^ (2018)	Loop mountain UM	168 (104 mile)	Mountain	10,000 positive altitude change	NR
Kishi et al^25^ (2024)	Point to point MUM, Race 1*^a^*	165	Mountain	9576 positive elevation	NR
	Point to point MUM, Race 2*^a^*	111	Mountain	6433 positive elevation	NR
Martin et al^33^ (2018)	From <36 hours to <60 hours	≥42.2	NR	NR	NR
Poussel et al^40^ (2015)	Loop mountain UM	168	Mountain	9600 elevation gain	NR

DDF, Diagonale des Fous; MUM, mountain ultramarathon; NR, not reported; TDB, Tour de Bourbon; UM, ultramarathon.

a Race 1= DDF, Race 2 = TDB.

### Sleep-Related Outcome Measures

Sleep was examined primarily through subjective reporting, with all included studies using ≥1 subjective sleep outcome measure.^3,10,12,13,22,23,25,33,40^ The outcome measures utilized were heterogenous. Five (56%) included studies opted to use a custom questionnaire for data collection, making this the approach used most in the studies in this review.^22,23,25,33,40^ Two studies (22%) used the Morningness-Eveningness Questionnaire (MEQ),^10,13^ the Pittsburgh Sleep Quality Index (PSQI),^37,40^ and the Epworth Sleepiness Scale (ESS).^10,13^ Outcome measures used in only a single (11%) included study were as follows: the Loughborough Daily Sleep Diary (LDSD),^3^ the Excessive Daytime Sleepiness Questionnaire (EDSQ),^13^ and the Karolinska Sleepiness Scale (KSS).^23^ Objective measurement of sleep was only measured in 1 (11%) study, where wrist-worn triaxial actigraphy was used.^22^ Finally, 1 (11%) study chose to record sleep timing during the race, but did not describe the method used.^12^ See Table 3 for sleep characteristic data.

**Table 3.** Sleep characteristics

Author (year)	Before race	Observed pattern*^a^*	During race	Observed pattern*^a^*
Benchertrit et al^3^ (2024)	Total sleep (hours) Week before Night before Rest time (hours) Week before Night before Sleep rating Week before Night before	7.29 (0.96) 6.78 (1.32) 8.31 (1.20) 8.26 (0.99) 3.68 (0.58) 3.45 (0.93)	Total sleep duration, minutes Number of naps taken Sleep deprivation, hours	1-15 1 >16
Daniel et al^10^ (2024)	Total sleep average, hours PSQI, n (%) Good Poor Sleep efficiency, % Sleep latency, minutes ESS	6.0 (1.2) 12 (32) 26 (68) 85.6 (14.6) 19.3 (14.8) 6.9 (4.9)	NR	NR
Doppelmayr et al^12^ (2005)	NR	NR	Total sleep duration, minutes Participants that slept, n (%) Number of naps taken	<120 2 (100) 2
Esteves dos Santos et al^13^ (2022)	PSQI Sleep efficiency, % Sleep latency, minutes ESS	4.66 (2.40) 88 (16) 13.88 (9.06) 9.55 (4.16)	NR	NR
Hurdiel et al^22^ (2015)	Rest time night before, hours	9.53 (0.59)	Total rest duration, minutes Total sleep duration, minutes Number of naps taken Sleep deprivation, hours:minutes	12 ± 17 9 ± 5 1.2 ± 1.4 46:38 ± 5:20
Hurdiel et al^23^ (2018)	Total sleep night before, hours	7.24 (0.92)	Participants that slept, n (%) Total sleep duration, minutes Nap duration, minutes Total sleep duration across all runners (n = 92), minutes	64 (69.6) 33.60 ± 19.20 14.40 ± 9.60 23.40 ± 22.20
Kishi et al^25^ (2024) Race 1	Planned implementation of a sleep strategy, n (%) Pre-race Intra-race Increase in average sleep duration, n (%) Decrease in average sleep duration, n (%)	544 (61.7) 358 (40.7) 414 (46.9) 500 (56.7) 9 (1.0)	Participants that slept, n (%) Total sleep duration, minutes Number of naps taken	735 (83.3) 76.1 ± 80.1 2.5 ± 2.1
Race 2	Planned implementation of a sleep strategy, n (%) Pre-race Intra-race Increase in average sleep duration, n (%) Decrease in average sleep duration, n (%)	129 (47) 47 (37.1) 33 (25.4) 133 (48.9) 3 (1.1)	Participants that slept, n (%) Total sleep duration, minutes Number of naps taken	144 (52.9) 27.2 ± 44.2 1.0 ± 1.3
All participants Race 1 and 2	Sleep duration, hours Implementation of sleep strategy, n (%) Pre-race Intra-race Increase in average sleep duration, n (%) Decrease in average sleep duration, n (%)	7.5 ± 1.0 672 (58.3) 450 (39) 484 (41.9) 633 (54.9) 12 (1.0)	Sleep duration, minutes (% of total naps)	<30 (82)
Martin et al^33^ (2018)	Average sleep time during training, hours Weekday Weekends Holidays ESS Implementation of sleep strategy, n (%)	6-7 7-8 8-9 8.9 ± 4.3 134 (21)	Participants slept, n (%) RL, 1 night RL, 2 nights RL, >2 nights Most common strategy	75 (16.4) 120 (55.6) 88 (94.6) Micro naps
Poussel et al^40^ (2015)	Implementation of sleep strategy, n (%) Naps Increased sleep time night before Sleep deprivation	266 (88) 124 (41) 110 (33) 33 (11)	Participants slept, n (%) Nap duration, minutes (% n)	86 (28) <10 (3), between 10 and 15 (5), between 15 and 30 (11), between 30 and 45 (2), between 45 and 60 (4), to > 60 (3)

Values are mean (SD) unless otherwise stated. ESS, Epworth Sleepiness Scale Score; NR, not reported; PSQI, Pittsburgh Sleep Quality Index; RL, race length.

a Definitions: Sleep rating, self-reported sleep quality on a 5-point Likert scale; PSQI, poor sleep (≥5); Sleep efficiency, adequate >85%; Sleep latency, poor sleep (>30 minutes); ESS, sleepiness >10; Sleep efficiency (%) = total time asleep/total time in bed.

### Performance Outcome Measures

The measurement of performance varied greatly throughout the included studies, of which 4 (44%) examined cognitive performance, 8 (88%) reported race finish time,^10,12,13,22,23,25,33,40^ and 1 reported on total distance covered.^3^ This difference was due to race type; the studies that reported on race time examined races with set distances, and the 1 study that examined total distance was a backyard-style race where the total laps, and therefore time, may be determined by dividing distance run by the lap length. Cognitive performance was measured differently across all the studies which examined it. One study used a modified version of the Automated Neurophysiological Assessment Metrics-General Neurophysiological Screening (ANAM-GNS) cognitive testing battery.^3^ This battery used 3 tasks to assess language control, problem solving, concentration, attention, reaction time, and executive function. The tasks selected were the Stroop Task, 2 Choice Reaction Time (2CRT), and Tower Puzzle, and each looked at change in reaction time (ΔRT) and change in throughput (ΔTP).^3^ Doppelmayr et al^12^ used 2 assessments during the race to track cognitive performance, the Konzentrations-Leistungs-Test (KLT) and the D2 Aufmerkamkeits-Belastungtest (D2). The KLT involves simple arithmetic to assess cognitive performance while the D2 assessed attention and concentration using a 1-minute character finding task.^12^ Similar to the D2, the Digital Symbol Substitution Task (DSST), a 1-minute cognitive performance measure, was used to assess cognitive performance.^22^ Finally, a 10-minute laboratory-based testing protocol was used to assess psychomotor vigilance performance.^23^

### Effect of Pre-Event and Intra-Event Sleep on Race Performance

Three studies reported statistically significant associations between pre-event sleep and race performance outcomes.^10,13,40^ Daniel et al^10^ reported that race finishers (21.8 ± 15.4 minutes) had longer pre-race sleep latency than nonfinishers (12.5 ± 10.8 minutes), and the event had higher sleep latency, with a statistically significant difference and a medium effect size (*P* < 0.001, Hedges’ *g* = 0.64). Esteves dos Santos et al^13^ reported a strong negative correlation between sleep efficiency and race finish time (*r* = −0.834; *P* = 0.04), indicating that higher sleep efficiency was associated with faster completion. Poussel et al^40^ observed that runners who increased sleep time on the night before competition completed the event faster than runners who intentionally trained under sleep-deprived conditions (*P* = 0.03). No other included studies reported statistically significant relationships between pre-event sleep variables and race performance outcomes.

Four studies examined intra-event sleep and race performance. Overall, these studies suggested that sleeping during the race, or accruing greater total intra-race sleep, was associated with longer elapsed finish time. Poussel et al^40^ reported that runners in a 168 km event who slept during the event (n = 86, 28%) completed the race more slowly than those who did not sleep (*P* < 0.001); however, limited additional statistics were provided beyond significance testing.^40^ Hurdiel et al^22^ also examined a 168 km event and reported a strong association between race duration and intra-race sleep and rest duration (*R*^2^ = 0.68; *P* < 0.001). Martin et al^33^ examined ultramarathons exceeding 42.2 km, although race distances were not reported. When races were stratified by duration, moderate positive correlations were found between intra-race sleep duration and finish time in races lasting 36 to 60 hours and >60 hours, but no relationship in races <36 hours due to very low levels of intra-race sleep in those events.^33^ In contrast, Kishi et al^25^ reported a moderate positive correlation between total intra-race sleep duration and finish time across 165 km and 111 km events, including the 111 km event with a mean duration <36 hours. Table 4 summarizes the association statistics for each study.

**Table 4.** Correlation of sleep outcome measures with race performance

Author (year)	Race performance variable	Sleep outcome measure	Results*^a^*	Summary of significant findings
Benchertrit et al^3^ (2024)	Distance covered (158.5 ± 53.8 km)	Time asleep Night before 7-day average	*r* = −0.46 *r* = −0.03	No statistical significance between distance covered and sleep outcomes
		Time in bed Night before 7-day average	*r* = 0.07 *r* = 0.19	
		Sleep rating Night before 7-day average	*r* = −0.58 *r* = −0.49	
Daniel et al^10^ (2024)	Race finish time (hours): good sleepers (43.7 ± 8.4) vs poor sleepers (47.2 ± 7.3)	PSQI	ES = 0.75	No significant correlation but medium effect size in race finish time between good and poor sleepers
	Finisher 28 (74%) vs nonfinisher 10 (26%)	All pre-race: Total sleep time PSQI Sleep efficiency (%) Sleep latency (minutes) ESS	ES = 0.39 ES = 0.28 ES = 0.45 ES = 0.64** ES = 0.30	Significant correlation between finishers and sleep latency
Esteves dos Santos et al^13^ (2022)	Race finish time (hours) (7.8 ± 4.7)	Sleep efficiency pre-race (%)	*r* = −0.834*	Significant negative correlation between race finish time and sleep efficiency
Hurdiel et al^22^ (2015)	Race finish time (hours:minutes) (36:02 ± 7:18)	Actigraph data of rest intra-race	*R*^2^ = 0.68**	Longer race time correlated positively with longer race sleep duration
Hurdiel et al^23^ (2018)	Race finish time (hours) 40.74 ± 4.23	Sleep intra-race	*r* = 0.24*	Significant but small positive correlation with sleep during race and race finish time
Kishi et al^25^ (2024)	Race 1 finish time (hours) (49.9 ± 7.9)	Total sleep time intra-race	*r* = 0.37**	Positive correlation between total sleep time and race finish time
	Race 2 finish time (hours) (33.6 ± 5.3)	Total sleep duration intra-race	*r* = 0.30**	Positive correlation between total sleep time and race finish time
	All participants: Races 1 and 2	Total sleep duration intra-race	*r* = 0.44**	Overall positive correlation between total sleep time and race finish time
Martin et al^33^ (2018)	Race finish time (hours) <36	Sleep duration intra-race	*r* = 0.05	Positive correlation between sleep duration and race time for races >36 hours
	36-60	Sleep duration intra-race	*r* = 0.48*	
	>60	Sleep duration intra-race	*r* = 0.44**	

ES, effect size; ESS, Epworth Sleepiness Scale score; ns, not significant; PSQI, Pittsburgh sleep quality index; *r*, Pearson’s correlation coefficient; *R*^2^, variance in dependent vs independent variable.

a Definitions: Sleep rating: self-reported sleep quality on a 5-point Likert scale. ES: ≥0.2, small; ≥0.5, medium; ≥0.8, large. PSQI: poor sleep, ≥5. Sleep efficiency: adequate >85%. Sleep latency: Poor sleep, >30 minutes; ESS: Sleepiness >10; Sleep efficiency (%) = total time asleep/total time in bed.

* *P* < 0.05; **P < 0.001.

### Effect of Pre-Event Sleep on Cognitive Performance

Benchertrit et al^3^ examined the association between pre-event sleep and cognitive performance assessed before and after a race. A moderate negative correlation was observed between participants’ average sleep rating over the 7 days before the event and change in reaction time on the 2CRT (ΔRT: *r* = −0.64; *P* = 0.03).^3^ A moderate positive correlation was also found between the same sleep measure and change in throughput on the 2CRT (ΔTP: *r* = 0.61, *P* = 0.05).^3^ Hurdiel et al^22^ assessed cognitive performance during the Ultra Trail du Mont Blanc (UTMB) and reported a progressive decline over the course of the event. Postrace reaction time and error count worsened relative to pre-race scores.^22^ In a subsequent study, Hurdiel et al^23^ assessed cognitive performance in the 24 hours before the UTMB and at 8 timepoints during the race. Scores decreased as the race progressed, with DSST performance declining by 0.1 correct answers for each hour spent on course (*t*(87) = −2.94, *P* < 0.01).^23^ Sleep obtained before testing was a significant predictor of DSST performance (*t*(86) = −3.19, *P* < 0.01).^23^

### Perceived Sleep Quality, Sleepiness, and Race-Related Outcomes

Subjective sleep measures were reported by multiple studies.^3,10,13,23^ In 1 study, perceived sleep quality was compared with training frequency and race performance.^10^ Participants who reported good sleep trained, on average, 1 additional session per week compared with those who reported poor sleep (*P* < 0.001).^10^ While perceived sleep quality was not associated significantly with race time, training frequency was significantly associated with faster finish times (*r* = −0.59; *P* = 0.03).^10^ Notably, the mean ESS score of 9.55 (±4.16) reported by Esteves dos Santos et al^13^ approached the clinical threshold for excessive daytime sleepiness (≥10), suggesting that a meaningful proportion of ultramarathon runners in this cohort may have been experiencing clinically relevant sleepiness during their typical training period, before any race-related sleep disruption. Hurdiel et al^23^ assessed self-reported sleepiness using the KSS. Sleepiness remained low the day before the race (mean score, 2.52; SD, 1.36), at the start line (mean score, 1.89; SD, 0.97), and at the finish line (mean score, 2.78; SD, 2.10).^23^ Sleepiness increased significantly over time, rising by 0.1 scale units for each hour spent on course: t(91) = 8.55; *P* < 0.01.^23^

### Effect of Intra-Event Sleep on Cognitive Performance

Hurdiel et al^23^ reported that cognitive performance declined over the course of the UTMB and that greater sleep obtained on course was associated with additional reductions in cognitive scores. Specifically, DSST scores decreased by 2.7 points for each hour slept during the event (t = −2.32; *P* = 0.02).^23^ In contrast, Hurdiel et al^22^ reported no significant association between cognitive performance and rest obtained during the race.

Clinical signs of sleep deprivation were reported in the UTMB cohort. Hurdiel et al^22^ found 47% of participants demonstrated clinical signs including extreme sleepiness, difficulty keeping eyes open while running, transient amnesia, and loss of balance, with several safety incidents reported during the event.^34^ Similarly, Doppelmayr et al^12^ reported marked declines in cognitive test performance as time elapsed, with 25.25 hours between the final on-course cognitive test and the last time a participant slept. Consistent with these findings, Hurdiel et al^23^ also reported worsening cognitive performance as the event progressed. Table 5 summarizes the relationships between intra-race sleep and cognitive outcomes across included studies.

**Table 5.** Sleep outcomes and cognitive performance

Author (year)	Sleep duration during race	Cognitive outcome measures	Results	Summary of significant findings
Benchertrit et al^3^ (2024)	1-15 minutes (n = 2)	2CRTA (%)RTThPStroop TestLevel 3 ALevel 3 RTInterference scoreTower PuzzleMean RT2CRT Δ RT + SR*^b^* 2CRT Δ ThP + SR*^b^*	Δ 0 ± 2.5Δ 77 ± 68*Δ –17.0 ± 11.4** Δ –2.5 ± 2.8*Δ 91 ± 119*Δ –4.3 ± 5.6 Δ 517 ± 296*r = −0.64*r = 0.61*	RT ↑ postrace. ThP and Level 3 A interference score↓ postrace. Moderate negative association between the average sleep ratings over 7 days pre-race and the 2CRT RT. A moderate association between the average sleep ratings over 7 days and the 2CRT ThP.
	









	
Doppelmayr et al^12^ (2005)	<120 minutes	KLT D2	±10% variation until 160 km. Performance ↓15% at 195 km Sub A: 0-70 km (↓ 21%) 95 km (↑4.9%) 95-195 km (↓25%) 215 km (baseline) Sub B: 0-30 km (↓12%) 30-160 km (baseline) 160-215 km (↓16%)	↓ throughout the race, slight ↑ to or above baseline on Day 2 after a 60-120 minute nap on night 1.
Hurdiel et al^22^ (2015)	12 ± 17 minutes	10 minute psychomotor vigilance testing		↑ errors of omission, commission and mean response times postrace. Cognitive performance vs rest obtained - no correlation. Cognitive performance vs elapsed race time - no correlation.
		Errors of omission (pre vs post)	Δ 4.3 ± 6.1*	
		Errors of commission (pre vs post)	Δ 0.8 ± 0.45*	
		Mean response time	Pre: 289 ± 43ms Post: 359 ± 92 ms Δ 70 ± 101.6 ms*	
Hurdiel et al^23^ (2018)	33.60 ± 19.20 minutes (n = 64)	DSST (1 minute version)		Scores ↓ as intra-race sleep ↑
		Pre	36.34 ± 6.47 correct	
		Post	34.12 ± 8.69 correct	

Throughput is the number of correct responses per minute of available response time. 2CRT, 2 choice reaction time; A, accuracy; D2, Aufmerksamkeits-Belastungs-test; DSST, digital symbol substitution task; KLT, Konzentrations-Leistungs-Test; *r*, Pearson’s coefficient; RT, reaction time; SR^b^, sleep rating 7-day average; ThP, throughput; ↑, increase; ↓, decrease; Δ, change in pre- and postrace scores.

* *P* < 0.05; **P < 0.001.

## Discussion

This systematic review synthesized evidence on how pre-race and intra-race sleep strategies relate to physical (race or event) performance and cognitive performance in ultramarathon runners. To our knowledge, this is the first review to bring both outcome domains together in the ultramarathon setting, and the overall findings were supported by a generally low risk of bias across included studies. The main findings were that reduced sleep was associated with poorer physical and cognitive performance, greater intra-race sleep was associated with longer finish times, and greater pre-race sleep was associated with faster completion times.

Sleep deprivation was common during ultramarathon events and was associated with substantial cognitive impairment as races progressed.^3,10,12,13,22,23,25,33,40^ Across studies, increasing sleep loss and longer time on course were accompanied by clinical signs of sleep deprivation and worsening cognitive performance, including slower reaction time and poorer attention or vigilance.^3,12,22,23^ These findings are consistent with broader research showing that executive function can vary by chronotype and time of day, and that acute sleep loss can also impair physical performance across multiple exercise domains, including endurance.^9,14^ Evidence from nonathlete populations similarly demonstrates measurable cognitive deficits after sleep loss, particularly during transitions to night work.^43^ Overall, the findings in this review support an association between sleep deprivation during ultramarathons and reduced cognitive performance, with physical performance effects appearing more variable and context-dependent.

Importantly, runners appeared to recognize the relevance of sleep management to maintaining performance. Most runners appear to implement ≥1 sleep strategy either before or during their event, suggesting that sleep planning is already embedded in how many ultramarathon runners approach preparation and racing.^25,33,40^ This practical insight matters because it highlights that the question is often not whether runners will use sleep strategies, but which strategies are most useful, and under what race conditions.

Cognitive decline and accumulating fatigue may also have safety implications in ultra-endurance events, particularly when terrain, night running, and sustained decision-making demands are present. In a 90 km event, illness-related medical encounters affected 1 in 52 starters.^44^ Across 111 km and 165 km events, 80% of 1154 finishers reported sleep deprivation symptoms, including hallucinations and falls, and 22% perceived increased accident risk after the race.^25^ Fatigue related to travel or sleep deprivation has been linked with greater risk-taking behavior, impaired judgement, and poorer decision-making in sport settings.^9,46^ Similar patterns are reported in high-risk occupational groups, where fatigue is associated with safety-compromising behaviors and increased injury risk.^34^ Although direct evidence linking objectively measured cognitive impairment with safety outcomes in ultramarathon runners remain limited, these findings support the need for research that treats cognitive performance and safety as meaningful outcomes alongside race time. A predictive model of cognitive performance has been developed for offshore sailing events of similar duration and sleep disruption demands.^21^ A comparable ultramarathon-specific approach could help identify points of greatest cognitive vulnerability and guide more targeted intra-race sleep planning.

Across included studies, sleep obtained during an ultramarathon was generally associated with longer finish time. In a UTMB cohort, runners who did not sleep during the race finished faster than those who slept.^40^ In another UTMB study, more sleep obtained on course was linked with slightly longer finish times.^22^ Survey-based findings suggest this relationship becomes clearer as event duration increases, with sleep during races showing a moderate positive association with finish time in events lasting ≥36 hours, but little relationship in shorter races where few participants reported sleeping.^33^ Similar patterns have been reported in larger race cohorts where cumulative sleep during the race increased with the number of nights spent on course and was associated with longer finish times.^25^

This finding needs a careful interpretation that separates what is mechanically true from what is biologically or behaviorally informative. Intra-race sleep directly adds to elapsed race time because sleeping is time not moving, so it can worsen performance when performance is defined by finish time. At the same time, intra-race sleep is also a marker that the runner has been on course long enough for sleep pressure and fatigue to necessitate stopping. In practice, longer time on course both increases the need for sleep and increases the opportunity to sleep, which can strengthen the observed association between sleep duration and finish time.^22,25^ Other race- and athlete-level factors may also influence both sleep and performance. Longer distances and greater elevation gain may increase race duration, fatigue, and sleep pressure, while athlete experience, pacing and nutrition may affect the need or decision to sleep and the likelihood of faster completion. As these factors were not measured consistently or adjusted for across studies, the independent contribution of intra-race sleep to performance cannot be determined. This means the relationship should not be interpreted as evidence that sleeping is inherently a poor strategy, or that avoiding sleep is universally optimal. Rather, on-course sleep likely reflects a trade-off, giving up time to preserve alertness, reduce perceptual fatigue, and maintain the capacity to continue safely and effectively when sleep pressure becomes overwhelming.^22,25^

From a practical standpoint, these data suggest that intra-race sleep is best viewed as both a contributor to elapsed time and a signal of physiological limitation in prolonged events. For some runners, especially in multi-night races, sleep may function less as a performance enhancer and more as a strategy that enables event completion and reduces cognitive vulnerability during high-risk segments such as overnight running.^12,22,23,25^ Future studies should distinguish time stopped specifically for sleep from other nonmoving time, and account for race duration, pacing and runner ability, so the performance implications of deliberate sleep planning can be separated from sleep that occurs because the event has already become prolonged.^3,33^

Pre-race sleep extension was associated with faster completion times compared with deliberately training under sleep-restricted conditions.^40^ One included study also reported that finishers had longer sleep latency than nonfinishers.^10^ However, mean sleep latency in finishers remained <30 minutes, which is typically considered within normal range, and the authors suggesting finishers may be more accustomed to poorer-quality sleep and therefore less sensitive to sleep loss commonly experienced during ultramarathons.^10^ Sleep obtained before testing was also a strong predictor of cognitive performance during the event.^23^ These findings are consistent with broader sport research indicating that sleep is a key contributor to subsequent performance.^5^ However, the observational nature of the included studies prevents causal inference. Athletes who increase their pre-race sleep may also differ in training quality, experience, race preparation, or overall health, which may partly explain the observed associations. Controlled studies using objective sleep measures are therefore required before specific recommendations regarding pre-race sleep extension can be made.

Interestingly, despite evidence supporting pre-race sleep, some runners rated their sleep quality as poorest on the night before competition.^3,22,23^ Participants also reported similar levels of sleepiness the day before, at the start line, and at the finish line.^23^ This mismatch suggests that subjective fatigue or sleepiness may not reliably reflect cognitive or race performance outcomes in this context.^25,40^ Furthermore, the mean ESS score approaching the clinical threshold for excessive daytime sleepiness reported by Esteves dos Santos et al^13^ suggests that some runners may already carry a meaningful sleep debt during training, before any race-related disruption occurs. This has practical implications for pre-race sleep planning, as athletes who are chronically under-slept during training may have less capacity to benefit from sleep extension strategies in the days immediately preceding competition. This reinforces the value of objective monitoring and structured pre-race sleep planning rather than relying on perceived sleep quality alone.

Interpretation of these findings is also constrained by the methods used to measure sleep. All included studies used ≥1 subjective sleep measure, 5 used custom questionnaires, and only 1 used wrist-worn actigraphy.^3,10,12,13,22,23,25,33,40^ Subjective assessment remain informative because they capture athletes’ perceived sleep experiences, which may represent a different aspect of sleep from parameters derived through objective monitoring.^1^ However, comparisons with actigraphy indicate that self-reported sleep duration may differ substantially from objectively recorded values and may show only limited agreement.^16,29^ Short awakenings may also not be recalled the following morning, meaning that sleep fragmentation can be under-reported.^53^ Actigraphy enables repeated assessment of sleep-wake patterns in field settings, but its limited ability to distinguish quiet wakefulness from sleep may result in overestimation of sleep and underestimation of wake time.^32^ Future studies should therefore combine validated prospective self-report tools with objective monitoring to assess both perceived sleep and sleep-wake behavior before and during ultramarathons.

A key strength of this review was the inclusion of runners from diverse settings and event types, improving the relevance of findings across the ultramarathon population. The search strategy, study selection, and data extraction were completed by multiple reviewers, reducing the likelihood of selection bias. However, all included studies were observational, most used small samples, and many were affected by confounding factors that are difficult to control in ultramarathon settings. Race distance, elevation, athlete experience, pacing, nutrition and other aspects of race preparation were reported or adjusted for inconsistently, limiting assessment of the independent relationship between sleep and performance. One included study comprised only 2 participants,^12^ and, while the descriptive findings from this study were consistent with broader patterns observed across the review, the small sample size precludes any statistical interpretation and its contribution to the overall narrative synthesis should be interpreted with caution. In addition, no included study reporting event distance examined a race >217 km, limiting the generalizability of the findings to longer and multi-day ultramarathon events, where cumulative sleep pressure and opportunities for intra-race sleep may differ. The predominance of subjective sleep measures, including custom questionnaires, and retrospective study designs introduce potential recall and reporting bias and limits the accuracy of sleep duration and timing estimates. Finally, substantial heterogeneity in exposure definitions and outcome measures prevented meta-analysis and limits certainty in the strength and generalizability of outcomes.

## Conclusion

This review found that greater or extended pre-race sleep was associated with faster finish times, while better pre-race sleep was associated with higher cognitive performance during racing. However, the observational evidence does not establish that increasing pre-race sleep improves performance, and the reported relationships may be influenced by factors such as differences in training and experience. Sleep deprivation during ultramarathons was associated consistently with cognitive decline, particularly as time on course increased. Intra-race sleep was generally associated with longer finish times, reflecting both the direct addition of nonmoving time and the likelihood that longer events and greater fatigue increase the need and opportunity to sleep. Future research using objective sleep measures and controlled designs that account for race duration, pacing, nonmoving time, and potential confounding is needed to clarify which sleep strategies best balance race performance with cognitive functioning and safety during ultramarathon events.

## Supplemental Material

sj-docx-1-sph-10.1177_19417381261494498 – Supplemental material for Sleep Strategies and Performance Outcomes in Ultramarathon Runners: A Systematic Review Supplemental material, sj-docx-1-sph-10.1177_19417381261494498 for Sleep Strategies and Performance Outcomes in Ultramarathon Runners: A Systematic Review by Daniel A. Brown, Jason Parreira, Katie Spencer, Serena Skinner, Wataru Goto and Larissa Sattler in Sports Health

sj-docx-2-sph-10.1177_19417381261494498 – Supplemental material for Sleep Strategies and Performance Outcomes in Ultramarathon Runners: A Systematic Review Supplemental material, sj-docx-2-sph-10.1177_19417381261494498 for Sleep Strategies and Performance Outcomes in Ultramarathon Runners: A Systematic Review by Daniel A. Brown, Jason Parreira, Katie Spencer, Serena Skinner, Wataru Goto and Larissa Sattler in Sports Health
