# Supplementary material for: Sleep Strategies and Performance Outcomes in Ultramarathon Runners: A Systematic Review
Source: Sports Health. 2026 Oct 28:19417381261494498. Online ahead of print. doi: 10.1177/19417381261494498 (PMC13719688; doi:10.1177/19417381261494498)
Supplement: Supplementary file 1 [file sj-docx-1-sph-10.1177_19417381261494498.docx]

Supplementary File 1 – Search Strategies for all Databases

| **Database** | **Date searched** | **Search strategy** | **No of results** |
| --- | --- | --- | --- |
| Pubmed | 25/7/25 | "sleep management strategies"[Title/abstract] OR “sleep extension"[Title/abstract] OR "sleep management"[Title/abstract] OR nap[Title/abstract] OR sleep*[Title/abstract] OR ("Sleep Hygiene"[Mesh] OR "Sleep Duration"[Mesh] OR "Sleep Latency"[Mesh] OR "Sleep Stages"[Mesh] OR "Sleepiness"[Mesh]) AND (performance) AND (ultramarathon[Title/abstract] OR "ultra marathon"[Title/abstract] OR "ultra-marathon"[Title/abstract] OR ultrarun*[Title/abstract] OR "ultra run*"[Title/abstract] OR "ultra-run*"[Title/abstract] OR "ultra endurance run*"[Title/abstract] OR "ultra-endurance run*"[Title/abstract] OR ("Running"[Mesh:NoExp])) NOT (triathl*[title/abstract] OR cycling[title/abstract] OR mice[title/abstract] OR animal[title/abstract]) | 132 |
| Embase | 25/7/2025 | 'sleep management strategies':ti,ab OR 'sleep extension':ti,ab OR 'sleep management':ti,ab OR nap:ti,ab OR sleep*:ti,ab OR ('Sleep Hygiene'/exp OR 'Sleep Duration'/exp OR 'Sleep Latency'/exp OR 'Sleep Stages'/exp OR Sleepiness/exp) AND (performance) AND (ultramarathon:ti,ab OR 'ultra marathon':ti,ab OR ultra-marathon:ti,ab OR ultrarun*:ti,ab OR 'ultra run*':ti,ab OR ultra-run*:ti,ab OR 'ultra endurance run*':ti,ab OR 'ultra-endurance run*':ti,ab OR (Running/de)) NOT (triathl*:ti,ab OR cycling:ti,ab OR mice:ti,ab OR animal:ti,ab) | 274 |
| PsycInfo(OVID) | 25/7/25 | "sleep management strategies".ti,ab. OR "sleep extension".ti,ab. OR "sleep management".ti,ab. OR nap.ti,ab. OR sleep*.ti,ab. OR (exp "Sleep Hygiene"/ OR exp "Sleep Duration"/ OR exp "Sleep Latency"/ OR exp "Sleep Stages"/ OR exp Sleepiness/) AND (performance) AND (ultramarathon.ti,ab. OR "ultra marathon".ti,ab. OR ultra-marathon.ti,ab. OR ultrarun*.ti,ab. OR "ultra run*".ti,ab. OR ultra-run*.ti,ab. OR "ultra endurance run*".ti,ab. OR "ultra-endurance run*".ti,ab. OR (Running/)) NOT (triathl*.ti,ab. OR cycling.ti,ab. OR mice.ti,ab. OR animal.ti,ab.) | 121 |
| SPORTDiscus | 25/7/25 | (TI "sleep management strategies" OR AB "sleep management strategies") OR (TI "sleep extension" OR AB "sleep extension") OR (TI "sleep management" OR AB "sleep management") OR (TI "nap" OR AB "nap") OR (TI "sleep*" OR AB "sleep*") OR (DE "Sleep Hygiene" OR DE "Sleep Duration" OR DE "Sleep Latency" OR DE "Sleep Stages" OR DE "Sleepiness") AND ("performance") AND ((TI "ultramarathon" OR AB "ultramarathon") OR (TI "ultra marathon" OR AB "ultra marathon") OR (TI "ultra-marathon" OR AB "ultra-marathon") OR (TI "ultrarun*" OR AB "ultrarun*") OR (TI "ultra run*" OR AB "ultra run*") OR (TI "ultra-run*" OR AB "ultra-run*") OR (TI "ultra endurance run*" OR AB "ultra endurance run*") OR (TI "ultra-endurance run*" OR AB "ultra-endurance run*") OR (DE "Running")) NOT (TI "triathl*" OR AB "triathl*") OR (TI "cycling" OR AB "cycling") OR (TI "mice" OR AB "mice") OR (TI "animal" OR AB "animal")) | 64 |
| Web of Science | 25/7/25 | Title:  "sleep management strategies" OR "sleep extension" OR "sleep management" OR nap OR sleep* OR ("Sleep Hygiene" OR "Sleep Duration" OR "Sleep Latency" OR "Sleep Stages" OR Sleepiness)  AND  All fields:  (performance)  AND  All Fields:  (ultramarathon OR "ultra marathon" OR ultra-marathon OR ultrarun* OR "ultra run*" OR ultra-run* OR "ultra endurance run*" OR "ultra-endurance run*" OR (Running))  NOT  All Fields:  (triathl* OR cycling OR mice OR animal)  Query link:  https://www.webofscience.com/wos/woscc/summary/44f33fe8-0ddd-420e-8c66-d1f0fc1de43b-017078462d/relevance/1 | 84 |
| Total: | | | 675 |
