# Supplementary material for: Sleep Strategies and Performance Outcomes in Ultramarathon Runners: A Systematic Review
Source: Sports Health. 2026 Oct 28:19417381261494498. Online ahead of print. doi: 10.1177/19417381261494498 (PMC13719688; doi:10.1177/19417381261494498)
Supplement: Supplementary file 2 [file sj-docx-2-sph-10.1177_19417381261494498.docx]

Supplementary File 2 - Joanna Briggs Institute scores: Prospective Cohort and Cross-sectional studies

| **Author, Year** | | **JBI Critical appraisal tool** | **Questions** | | | | | | | | | | | **CAS** | **Overall score (%)** |
| --- | --- | --- | --- | --- | --- | --- | --- | --- | --- | --- | --- | --- | --- | --- | --- |
|  |  | **1** | | **2** | **3** | **4** | **5** | **6** | **7** | **8** | **9** | **10** | **11** |  |  |
| Benchertrit et al., 2024[4] | | Prospective cohort | NA | NA | 1 | 1 | 1 | 1 | 1 | 1 | 1 | NA | 1 | 8 | 100 |
| Doppelmayr et al., 2005[36] | | Prospective cohort | NA | 1 | 0 | 1 | 0 | 1 | 1 | 1 | 1 | NA | 1 | 7 | 78 |
| Hurdiel et al., 2015[34] | | Prospective cohort | NA | 1 | 1 | 1 | 1 | 1 | 1 | 1 | 1 | 1 | 1 | 10 | 100 |
| Hurdiel et al., 2018[35] | | Prospective cohort | NA | 1 | 1 | 1 | 1 | 1 | 1 | 1 | 1 | 1 | 1 | 10 | 100 |
| Daniel et al.,   2024[37] | | Cross-sectional | 1 | 1 | 1 | 1 | 0 | 0 | 1 | 1 |  |  |  | 6 | 75 |
| Esteves dos Santos et al., 2022[40] | | Cross-sectional | 1 | 1 | 1 | 1 | 1 | 0 | 1 | 1 |  |  |  | 7 | 88 |
| Kishi et al., 2024[38] | | Cross-sectional | 1 | 1 | 1 | 1 | 1 | 0 | 0 | 1 |  |  |  | 6 | 75 |
| Martin et al., 2018[14] | | cross-sectional | 1 | 1 | 0 | 0 | 1 | 0 | 1 | 1 |  |  |  | 5 | 63 |
| Poussel et al., 2013[39] | | Cross-sectional | 1 | 1 | 0 | 0 | 1 | 0 | 1 | 1 |  |  |  | 5 | 63 |

Legend: CAS = Critical appraisal score; 1 = Yes; 0 = No.
